# Dialysis Arteriovenous Fistula Causing Subclavian Steal Syndrome in the Absence of Subclavian Artery Stenosis

**DOI:** 10.1155/2015/720684

**Published:** 2015-04-16

**Authors:** Eesha Maiodna, Sudheer Ambekar, Jeremiah N. Johnson, Mohamed Samy Elhammady

**Affiliations:** Department of Neurological Surgery, University of Miami School of Medicine, Miami, FL 33136, USA

## Abstract

We present a rare cause of subclavian steal syndrome secondary to a dialysis arteriovenous fistula (AVF). A 69-year-old female with end-stage renal disease presented with ataxia and recurrent fainting spells. Angiography revealed normal subclavian arteries bilaterally, a right VA origin occlusion, and an apparent left VA origin occlusion. However, carotid artery angiography demonstrated flow through the posterior communicating artery with retrograde filling of the basilar artery and left VA to its subclavian origin. Repeat left subclavian arteriography during external compression of the AVF demonstrated normal antegrade left VA flow. The AVF was subsequently ligated resulting in complete symptom resolution.

## 1. Introduction

Subclavian steal is the reversal of flow in a vertebral artery (VA) due to stenosis or extrinsic compression of the ipsilateral innominate or subclavian artery proximal to the VA origin. Although subclavian artery stenosis is typical, subclavian steal syndrome may also occur with a normal subclavian artery. We present a rare cause of subclavian steal syndrome secondary to a dialysis arteriovenous fistula (AVF). To our knowledge, this is the third case reported in the literature.

## 2. Case Report

A 69-year-old female with diabetes, chronic hypertension, and end-stage renal disease presented with progressive ataxia and recurrent fainting spells. Three months prior to presentation she had undergone a dialysis AV fistula with left brachial artery to basilic vein transposition (BVT). The patient also reported several left upper extremity symptoms including numbness, tingling, cold fingers, cramping, and decreased fine motor skills, as well as bluish discoloration particularly on the days of dialysis. On examination, the left radial pulse was not palpable, the left hand was cold to touch, and handgrip was minimally weak. The patient had impaired left sided finger-to-nose testing and an abnormal wide-based gait.

**Figure 1:**
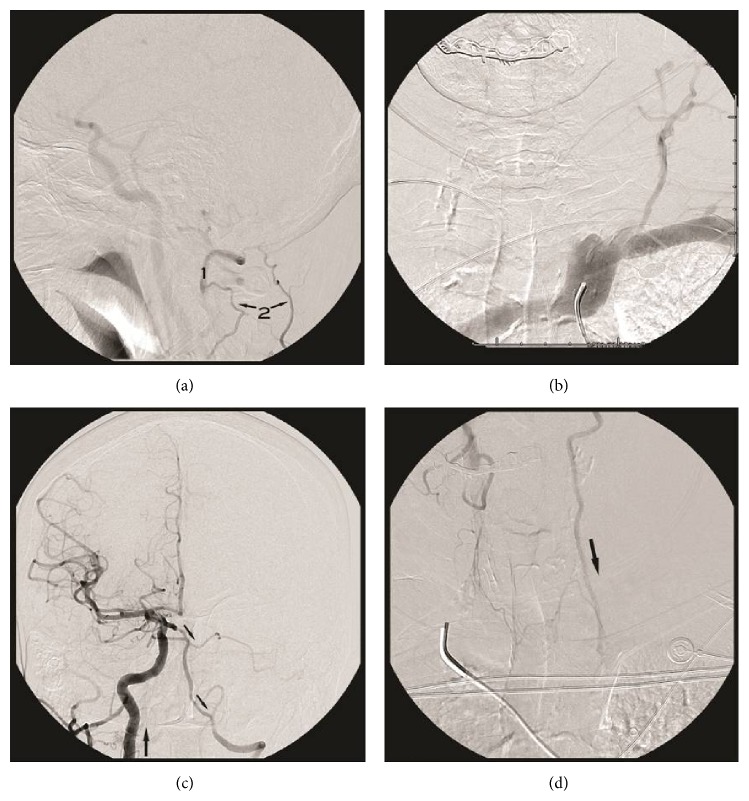
(a) Lateral right subclavian artery angiogram (cranial view) demonstrating occlusion of the right VA with distal reconstitution (1) through muscular branches of the right ascending cervical artery (2). (b) Left subclavian artery angiogram demonstrating normal subclavian artery caliber and a stump at the left vertebral origin with no antegrade VA flow. (c and d) Anteroposterior right common carotid angiogram (cranial and cervical views, resp.) demonstrating flow through the p-comm. artery with retrograde filling of the basilar and left VA down to the level of the subclavian artery.

**Figure 2:**
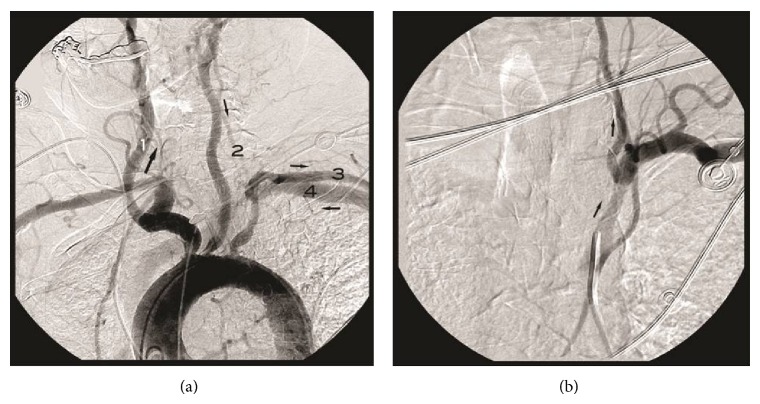
(a) Aortic arch angiogram demonstrating the overall hemodynamics. There is occlusion of the right VA origin and antegrade flow through the right common carotid artery (1) with reversal of flow in the left VA (2) down to the left subclavian artery (3). Note the early antegrade flow through the left subclavian artery (3) and the early filling of the left subclavian vein (4) indicative of the high flow through the dialysis AVF. (b) Anteroposterior left subclavian angiogram with occlusion of the AVF shows reestablishment of normal antegrade flow through the left vertebral artery.

Magnetic Resonance Imaging with angiography (MRI/MRA) demonstrated a chronic cerebellar infarct with a hypoplastic right vertebral and basilar artery. The patient was referred to our service for further evaluation with digital subtraction angiography. First, a right subclavian artery angiogram was performed which showed occlusion of the right VA at its origin with distal reconstitution at the level of the C1 vertebral body through muscular branches of ascending cervical artery (Figure 1(a)). A left subclavian angiogram demonstrated a normal subclavian artery caliber and a stump at the origin of the left VA without distal flow (Figure 1(b)). A right common carotid angiogram demonstrated flow though the posterior communicating artery with retrograde filling of the basilar artery and left VA to its subclavian origin (Figures 1(c) and 1(d)). We realized that the left VA was not occluded, and the reversal of flow seen was most likely related to steal from the patient's left arm dialysis AVF. An aortic angiogram demonstrated the overall hemodynamics (Figure 2(a)). A blood pressure cuff was then inflated on the left upper extremity to occlude flow to the fistula. A repeat left subclavian angiogram demonstrated reestablishment of normal antegrade flow through the left VA (Figure 2(b)). The patient was treated surgically by ligating the fistula, which resulted in symptom resolution.

## 3. Discussion

Subclavian steal phenomenon (SSP) is a benign vascular hemodynamic condition characterized by reversal of flow in the VA due to stenosis or occlusion of ipsilateral innominate or subclavian artery proximal to the origin of VA. Subclavian steal syndrome (SSS) is the term used for a symptomatic steal phenomenon. The first report of a subclavian steal phenomenon was by Contorni and colleagues in 1960 [1]. The following year, Reivich et al. described a case of symptomatic subclavian steal phenomenon. C. M. Fisher coined the term “subclavian steal syndrome” in the editorial discussion of the classic report by Reivich et al. [2].

The estimated prevalence of subclavian steal phenomenon is 0.6–6.4% [3–5]. The most common etiology of subclavian steal is atherosclerotic stenosis of the subclavian artery. It is more commonly seen on the left side and is believed to be a result of the acute angle of origin of the left subclavian artery, which may result in local turbulence of blood flow and subsequent atherogenesis [3–6]. Other rare causes include large-vessel vasculitis (e.g., Takayasu or giant cell arteritis), extrinsic compression (costoclavicular syndrome), iatrogenic stenosis (e.g., radiation-induced), and congenital vascular anomalies [7].

Symptomatic subclavian steal generally occurs during exercise or use of the ipsilateral upper extremity and can manifest with the symptoms of vertebrobasilar insufficiency, ipsilateral upper extremity ischemia, or, rarely, coronary ischemia. In addition to the severity of subclavian stenosis, the adequacy of collateral cerebral blood flow as well as the presence and location of associated extracranial vascular stenosis represents a major determining factor for the development of symptoms and reversal of flow in the VA [3, 6, 8].

The diagnosis of subclavian steal can be suspected clinically by the presence of a subclavian artery bruit and a difference in systolic blood pressure of more than 20 mmHg between the arms [4, 5, 9]. Although digital subtraction angiography is the gold standard, color doppler flow imaging and continuous wave ultrasonography have been reported as effective noninvasive, cost-effective, and sensitive screening tools [3, 8, 10–12].

Dialysis AV fistulae are an extremely rare cause of SSS. Arteriovenous fistulae of the extremities can be classified as small or large if the diameter of the fistula is less or greater than 75% of the arterial lumen, respectively. Surgically created fistulas are of the large type [13]. They act as low-pressure, low-resistance, high-flow systems diverting considerable volume of blood [12].

According to the National Kidney Foundation DOQI guidelines for vascular access, a BVT may have higher incidence of subclavian steal and arm swelling than other fistula types [14]. Dialysis-associated steal syndrome (DASS) or distal hypoperfusion ischemic syndrome (DHIS) is a complication caused by arterial insufficiency distal to the dialysis access owing to diversion of blood into the fistula or graft; its pathophysiology is similar to SSP. Although reversal of flow has been documented in up to 73% of cases after radiocephalic fistula and in up to 91% of cases after brachial artery axillary vein graft [15], symptomatic ischemia is seen in only 10–25% of brachiocephalic and basilic AVFs [16, 17].

Our patient had a BVT, which is a large type AVF involving a large caliber artery and vein, and presented with symptoms of vertebrobasilar insufficiency and ischemic symptoms of the hand. Although the synergistic effect of mild subclavian artery stenosis and dialysis AV fistula as a cause of subclavian steal has been reported by Bron et al. [18], to our knowledge there are only 2 reported cases of SSS secondary to a dialysis AVF in the absence of subclavian stenosis [19, 20]. Boettinger et al. reported a case of 59-year-old man with chronic renal failure presenting with acute left homonymous hemianopsia as a result of a SSS due to an AVF [19]. In another case reported by Schenk III, a 28-year-old dialysis-dependent man presented with symptoms of vertebrobasilar insufficiency secondary to a brachiocephalic AVF. There was reversal of symptoms and reestablishment of antegrade flow in the VA after surgical reduction of flow through the AVF by a banding technique [20].

## 4. Conclusion

Although subclavian steal phenomenon is a benign vascular condition, subclavian steal syndrome is clinically significant and may result in symptoms of vertebrobasilar insufficiency and ipsilateral hand ischemia. Atherosclerosis is the most common etiology; however, other rare causes must be considered including high-flow dialysis AVFs. Recognition of this rare iatrogenic cause of SSS is important as it can lead to permanent neurological injury but is promptly reversed by decreasing AVF flow.
